# In Silico Designing of a Multitope Vaccine against *Rhizopus microsporus* with Potential Activity against Other Mucormycosis Causing Fungi

**DOI:** 10.3390/cells10113014

**Published:** 2021-11-04

**Authors:** Mohamed A. Soltan, Muhammad Alaa Eldeen, Nada Elbassiouny, Hasnaa L. Kamel, Kareem M. Abdelraheem, Hanaa Abd El-Gayyed, Ahmed M. Gouda, Mohammed F. Sheha, Eman Fayad, Ola A. Abu Ali, Khalid Abd El Ghany, Dalia A. El-damasy, Khaled M. Darwish, Sameh S. Elhady, Ashraf E. Sileem

**Affiliations:** 1 Department of Microbiology and Immunology, Faculty of Pharmacy, Sinai University, Ismailia 41611, Egypt; hasnaa.kamel@su.edu.eg; 2 Cell Biology, Histology & Genetics Division, Zoology Department, Faculty of Science, Zagazig University, Zagazig 44519, Egypt; dr.muhammadalaa@gmail.com; 3 Department of Pharmacology and Toxicology, Faculty of Pharmacy, Sinai University, Ismailia 41611, Egypt; nada.elbasuny@su.edu.eg; 4 Department of Biochemistry, Faculty of Pharmacy, Zagazig University, Zagazig 44519, Egypt; kareemabdelraheem92@gmail.com (K.M.A.); drhanaa987@gmail.com (H.A.E.-G.); 5 Department of Pharmacy Practice, Faculty of Pharmacy, Zagazig University, Zagazig 44519, Egypt; amgoda@pharmacy.zu.edu.eg; 6 Department of Biochemistry, Faculty of Pharmacy, Suez Canal University, Ismailia 41522, Egypt; mohamed.f.sheha@gmail.com; 7 Department of Biotechnology, Faculty of Sciences, Taif University, P.O. Box 11099, Taif 21944, Saudi Arabia; e.esmail@tu.edu.sa; 8 Department of Chemistry, College of Science, Taif University, P.O. Box 11099, Taif 21944, Saudi Arabia; O.abuali@tu.edu.sa; 9 Egyptian Drug Authority, Giza 35521, Egypt; masterkhalid11@gmail.com; 10 Department of Microbiology and Immunology, Faculty of Pharmacy, Egyptian Russian University, Cairo 11829, Egypt; daliadamsy@eru.edu.eg; 11 Department of Medicinal Chemistry, Faculty of Pharmacy, Suez Canal University, Ismailia 41522, Egypt; khaled_darwish@pharm.suez.edu.eg; 12 Department of Natural Products, Faculty of Pharmacy, King Abdulaziz University, Jeddah 21589, Saudi Arabia; ssahmed@kau.edu.sa; 13 Department of Chest Diseases, Faculty of Medicine, Zagazig University, Zagazig 44519, Egypt; Sileem.ashraf@yahoo.com

**Keywords:** mucormycosis, immunoinformatics, epitope mapping, multitope vaccine, COVID-19 pandemic

## Abstract

During the current era of the COVID-19 pandemic, the dissemination of Mucorales has been reported globally, with elevated rates of infection in India, and because of the high rate of mortality and morbidity, designing an effective vaccine against mucormycosis is a major health priority, especially for immunocompromised patients. In the current study, we studied shared Mucorales proteins, which have been reported as virulence factors, and after analysis of several virulent proteins for their antigenicity and subcellular localization, we selected spore coat (CotH) and serine protease (SP) proteins as the targets of epitope mapping. The current study proposes a vaccine constructed based on top-ranking cytotoxic T lymphocyte (CTL), helper T lymphocyte (HTL), and B cell lymphocyte (BCL) epitopes from filtered proteins. In addition to the selected epitopes, β-defensins adjuvant and PADRE peptide were included in the constructed vaccine to improve the stimulated immune response. Computational tools were used to estimate the physicochemical and immunological features of the proposed vaccine and validate its binding with TLR-2, where the output data of these assessments potentiate the probability of the constructed vaccine to stimulate a specific immune response against mucormycosis. Here, we demonstrate the approach of potential vaccine construction and assessment through computational tools, and to the best of our knowledge, this is the first study of a proposed vaccine against mucormycosis based on the immunoinformatics approach.

## 1. Introduction

Mucormycosis is an invasive fungal infection caused by a diverse group of fungi belonging to the order Mucorales [1]. The major ways of infection in humans are through the inhalation of sporangiospores and the ingestion of contaminated food with Mucorales spores [2]. After analysis, 11 genera and approximately 27 species in the order Mucorales were estimated as mucormycosis causative agents to humans, where *Rhizopus* came at the top of this list as the most common mucormycosis causative genus around the world [3].

In mucormycosis, various disease manifestations are seen, such as rhinocerebral mucormycosis, which affects the sinuses and the brain; pulmonary mucormycosis, which affects the lung and lead to breathing disturbances and cough; cutaneous mucormycosis, which lead to ulcers and blisters at the site of infection; gastrointestinal mucormycosis, which is more common in neonates than adults; and finally, disseminated mucormycosis, that affect more than one organ in the infected person [4]. The overall mortality rate of mucormycosis was reported to be between 46% and 54% [5].

The traditional risk factors of mucormycosis are diabetes mellitus, hematological malignancy, chronic kidney disease, and trauma, where the latter is a major risk factor for cutaneous mucormycosis [6]. Recently, with the outbreak of COVID-19, many cases of mucormycosis have been reported in people infected with COVID-19 around the world and especially in India [7]. This correlation was attributed to the excellent environment of high glucose, low oxygen, and decreased fighting activity of white blood cells in patients infected with COVID-19, which made it easy for Mucorales spores to germinate [8]. COVID-19 patients who required a ventilator or prolonged hospital admission had a high chance of fungal co-infection. Moreover, corticosteroid utilization during the COVID-19 course of treatment would inhibit immune responses, allowing mucormycosis infections that can manifest through various symptoms, starting from nasal congestion and rhinorrhea and moving to loss of vision and finally, fatal tissue necrosis. The management of mucormycosis requires several steps, starting from early diagnosis, the reversal of environmental conditions which facilitate the germination process, the surgical removal of damaged tissues, and the administration of a suitable antifungal agent [9]. While amphotericin B, posaconazole, and isavuconazole are categorized as the most active antifungal agents against Mucorales, their activity remains suboptimal [10]. Before the current time of the COVID-19 pandemic, which facilitates the spreading of mucormycosis, the United States health care system cost about $50 million per year for the management of mucormycosis cases, leading to the question about the economic value of developing a vaccine against mucormycosis, where analysis and studies proved the requirement of an effective vaccine that will reduce the infection rate and mortality in a cost-effective manner [11].

Few studies have tried to design an effective vaccine against mucormycosis through conventional approaches. One of these studies recommended the potential application of heat-killed *Saccharomyces cerevisiae* to protect mice from mucormycosis [12]. During the last twenty years, humanity experienced a massive progression in sequencing techniques, resulting in tremendous data on the genome and proteome of many organisms. This progression was accompanied by a development in the computational tools that can handle and analyze these data [13]. Based on that, the field of vaccine development witnessed the initiation of the immunoinformatics field as a modern approach that can predict vaccine candidates against harmful microorganisms [14]. The tools of immunoinformatics were applied to construct putative epitope vaccines against many microorganisms such *Staphylococcus aureus* [15], *Moraxella catarrhalis* [16], the Zika virus [17], *Candida albicans* [18], and *Pseudomonas aeruginosa* [19]. In addition to that, a biological validation has been performed for the multitope vaccine against *Trichinella spiralis* [20] and uropathogenic *Escherichia coli* [21], and the results were promising. The current study aimed to identify the virulence proteins of mucormycosis-causing fungi for B- and T-cell epitope prediction to construct a chimeric epitope vaccine against mucormycosis and to analyze the antigenicity and the reactivity of the final vaccine construct through computational tools.

## 2. Materials and Methods

**Figure 1:**
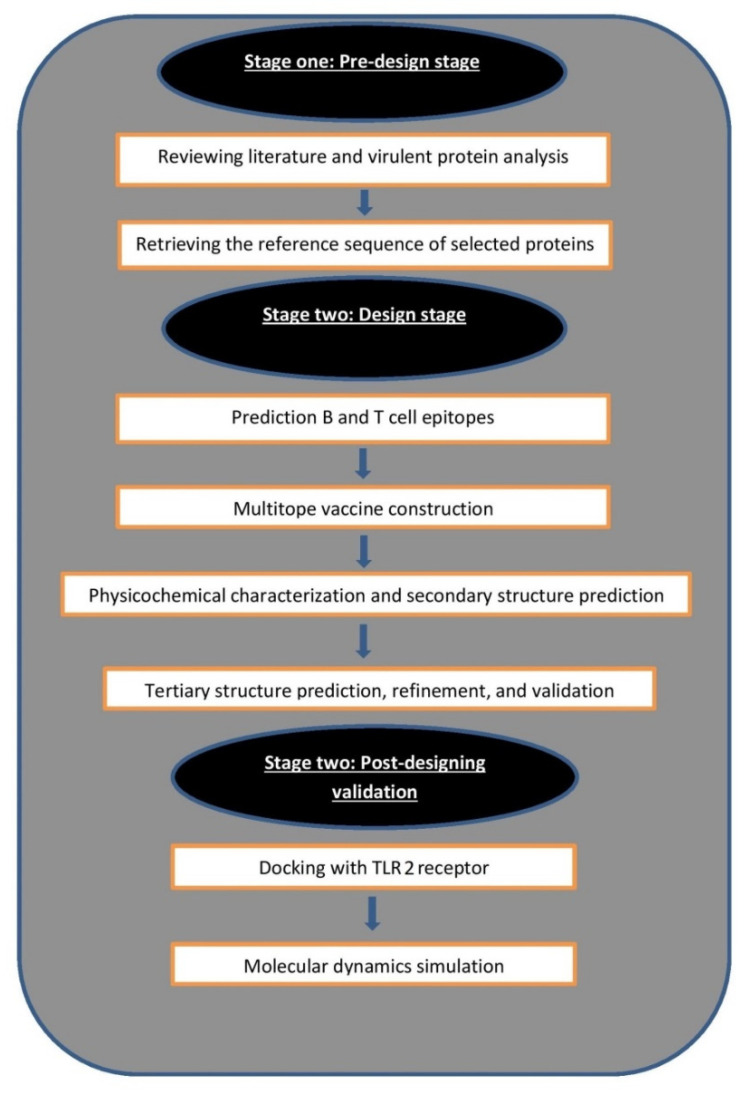
Graphical representation shows the major steps for constructing a multitope vaccine against mucormycosis.

A graphical summary of the current study flow of work is demonstrated in Figure 1.

### 2.1. Selection of Proteins

While *Rhizopus arrhizus* is the most common causative agent around the world and consequently the basic target of the current study, it has no proteome sequence of a reference strain deposited on NCBI, and investigation of uploaded proteome sequences showed that all proteins were named hypothetical protein; therefore, the whole proteome of *Rhizopus microsporus* ATCC 52813 reference strain (high-quality, fully authenticated certified reference material) was retrieved from NCBI with the GenBank assembly accession number GCA_002708625.1. Based on mentioned common virulent proteins in mucormycosis-causing fungi [22], we analyzed these proteins based on their antigenicity and subcellular localization. Antigenicity assessment was carried through Vaxigen v2.0 [23], where proteins with antigenicity score more than 0.5 were considered antigenic. We planned to select protein candidates to pass to the epitope-mapping stage, and the criteria of the passed proteins were the following: they must have an antigenicity score of more than 0.5 and be presented on the cell wall or secreted to the outer media in order for them to have direct contact with the infected host, and finally, conserved in various species of mucormycosis-causing fungi so that the designed chimeric vaccine would have a cross-reactivity against them and consequently compensate the principle of obtaining the protein candidates’ sequence from *Rhizopus microspores,* which is not the most common mucormycosis-causative fungi.

### 2.2. Prediction of B and T Cell Epitopes

As a primary step before epitope prediction, selected proteins were analyzed for the presence of signal peptides through SignalP-5.0 server [24] which estimates the exact location of signal peptide cleavage sites in submitted proteins. The second step was the submission of mature proteins to the Immune Epitope Database (IEBD) [25]. This database provides several approaches for MHC-I assessment, and the current study adopted NetMHCpan EL 4.0 as a method for MHC-I peptide prediction, and this was the recommended one by the server. The reference set of HLA alleles was utilized, along with the mentioned assessment method, due to the ability of this list to cover commonly shared binding specificities, resulting in 97% of population coverage [26]. The second assessment was for MHC-II epitopes, and this was also run through the recommended server’s method (2.22 prediction method). To cover a high percentage of the population, the full HLA reference set was used with the recommended prediction method [27]. Following that, MHC-II epitopes that obtained high estimation scores were submitted to INF prediction server to evaluate their ability to induce INF-γ [28]. The last estimation on this server was the prediction of B-cell epitopes which was performed through bepipred linear epitope prediction method [29]. In order to confirm the affinity of the selected peptides toward their respective alleles, each peptide three-dimensional structure was estimated via PEP FOLD 3 web server [30]; concurrently, three-dimensional structure of HLA-A*11:01 (PDB ID 6JP3) and HLA-DRB1*04:01 (PDB ID 5JLZ) was obtained from the protein data bank for MHC-I and MHC-II epitopes, respectively, to act as receptors for single epitope docking estimation. Docking was analyzed through AutoDock Vina [31]. Peptides that passed the above-mentioned filtration stages were analyzed for the last time before multitope assembly for their conservation in multiple mucormycosis-causing fungi to generate a vaccine with potential cross-reactivity. This analysis was performed through BLASTp.

### 2.3. Multitope Vaccine Construction

Four basic components were assembled to construct the current study’s predicted multitope vaccine. First of all, β-defensin adjuvant sequence was added, then the recommended single epitopes from the previous stage of analysis. These epitopes were linked together with amino acid linkers, which represented the third component of the constructed vaccine, while the fourth and last component to be added was PADRE sequence [32]. The complete vaccine construct was analyzed for many properties. The antigenicity was predicted via VaxiJen v 2.0 [23], while the allergenicity was estimated by AlgPred server [33]. The final assessment was for the toxicity, and we employed ToxinPred web server for this purpose [34].

### 2.4. Assessment of Vaccine’s Solubility, Physicochemical Features, and 2^ry^ Structure

Estimation of the constructed vaccine solubility upon overexpression in *Escherichia coli* was done via SOLpro online server [35], while the assessment of the physicochemical characteristics was performed through ProtParam tool [36]. Lastly, the vaccine’s secondary structure predicted was performed by PSIPRED server [37].

### 2.5. Vaccine 3D Structure Prediction and Validation

3Dpro online server was employed for the prediction of the vaccine 3D structure [38]. The server applies multiple models to come up with a predicted model having the lowest possible energy and a high grade of stability. Following that, the generated 3D model was refined through uploading on GalaxyRefine server [39]. Lastly, and to evaluate the generated 3D structure and the refinement process, Ramachandran plot analysis [40] and ProSA [41] were employed to analyze the original predicted structure and the refined one.

### 2.6. Vaccine Disulfide Engineering

Modification of the protein structure through the addition of disulfide bonds is important as these bonds play a significant role in improving the stability of a protein; to achieve that, disulfide by design 2.0 [42] was chosen to sign these bonds to the validated vaccine three-dimensional structure.

### 2.7. Docking Analysis between Predicted Vaccine 3D Structure and TLR-2

In the field of in silico drug design, docking studies are of great importance as they predict the possible complexes that may generate from the binding between a ligand and its respective receptor and the numerical value of the ligand–receptor binding energy provides an estimation of the affinity between the complex components [43]. Neutrophils have a vital role in combating mucormycosis and as a result of neutrophil spore interaction, excessive expression of toll-like receptor (TLR) occurs [44]. Furthermore, *Rhizopus hyphae* were found to induce TLR-2 expression [45]; thus, TLR-2 (PDB id: 2Z7X)’s 3D structure was retrieved from the protein databank and uploaded into the ClusPro 2.0 server to act as a receptor for the docking study [46], while the ligand of this analysis was the predicted vaccine 3D structure. This server works by estimating a large number of possible complex orientations and finally, by providing the conformation with the highest predicted stability.

### 2.8. Normal Mode Analysis

iMODS server [47] was employed to perform normal mode analysis for the complex between the designed vaccine 3D structure and TLR-2. The server analyzes the collective motions of the vaccine-receptor complex using normal mode analysis in internal coordinates [48].

### 2.9. Molecular Dynamics Simulation

The multitope vaccine/TLR-2 docked complex was chosen as starting coordinates for a 50-nanosecond all-atom molecular dynamics (MD) simulation using GROMACS-2019 software package (GNU General Public License http://www.gromacs.org) accessed on 23 September 2021 [49]. The CHARMM36m force field was selected in many studies that applied MD [50,51]. The docked complex was solvated within a cubic box of the TIP3P water model under periodic boundary conditions implementation [52]. The MD simulations were conducted over three conventional stages; one-staged minimization, double-staged equilibration, and production [53,54]. The production stage involved 50 ns MD simulation runs under constant pressure (NPT ensemble) while using the Particle Mesh Ewald (PME) algorithm for computing the long-range electrostatic interactions [55]. All covalent bond lengths, including hydrogens, were modeled under the implemented linear constraint LINCS method [55]. Both Coulomb’s and van der Waals non-bonded interactions were truncated at 10 Å using the Verlet cut-off scheme [56].

Computing comparative analysis tools, including RMSD, RMSF, radius of gyration (Rg), and solvent-accessible surface area (SASA) were performed through analyzing the MD trajectories using the GROMACS built-in tools. Binding-free energy between the ligand and protein was estimated via the Molecular Mechanics/Poisson–Boltzmann Surface Area (MM/PBSA) using GROMACS “g_mmpbsa” module [57]. To represent the ligand-protein conformational analysis, the Schrödinger-Pymol graphical software was employed.

### 2.10. Reverse Translation and Codon Adaptation

The final step of the current computational study is the reverse translation and codon adaptation for the vaccine amino acid sequence to be expressed in *E. coli* k-12, as an expression that will be the first step in the wet-lab validation of the currently proposed vaccine, and for this purpose, JCAT server [58] was employed.

### 2.11. Immune Simulation of the Chimeric Peptide Vaccine

The stimulated immune response for the designed multitope vaccine was predicted computationally through C-ImmSim server [59]. We investigated the immune response after the administration of three multitope vaccine injections in four weeks intervals. This technique represents a prime-booster-booster approach to achieve a long-lasting immune response.

## 3. Results

### 3.1. Nomination of Two Proteins as Vaccine Candidates

We planned to select protein candidates (from virulent proteins mentioned in [19]) with antigenicity scores of more than 0.5 and to have direct contact with the human host after infection. Firstly, virulent proteins were obtained from the proteome of *Rhizopus microsporus* ATCC 52813 and analyzed according to selection criteria (Table 1). Serine protease (SP), spore coat protein (CotH), and calcium/calmodulin-dependent protein kinase passed the antigenicity score filtration step and analyzed for their subcellular localization, where SP was found to be a secretory protein and CotH was a surface-exposed protein.

### 3.2. Prediction of B Cell Epitopes

**Figure 2:**
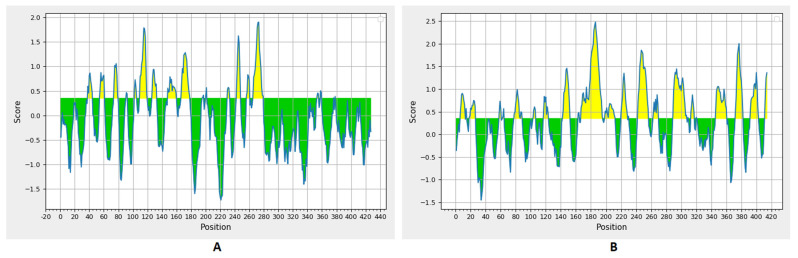
Bepipred linear epitope prediction for CotH (**A**) and SP (**B**) proteins; the yellow section represents the B epitope part of each protein.

The B-cell epitopes had a threshold value of 0.35 (Figure 2). There were 19 and 23 predicted epitopes for CotH and SP, respectively. This list was downsized by selecting epitopes sized between 8 and 18 peptides (Table 2), and finally, the top two epitopes, according to their antigenicity score for each of the protein candidates, were selected to construct the multitope vaccine.

### 3.3. Prediction of MHC-I and MHC-II Epitopes

For MHC-I epitopes, 22,599 and 21,897 epitopes were predicted for CotH and SP, respectively, and the percentile rank of these predicted epitopes ranged from 0.01 to 100. In order to shorten the list for epitope selection, only epitopes with a percentile rank lower than two were analyzed to choose the best candidates between them. The reason for taking peptides with a percentile rank lower than two is that the smaller the percentile rank, the more binding affinity with the respective allele. Other factors were considered in the selection step, such as the antigenicity score and the number of reacting alleles (Table 3). Regarding MHC-II epitopes, there were 11,151 and 10,800 predictions for CotH and SP, respectively; again, the percentile rank, antigenicity score, number of reacting alleles, and the epitope ability to induce interferon gamma were the criteria for the best candidates selection (Table 4).

### 3.4. Molecular Docking of T Cell Epitopes and Assessment of Selected Epitopes Conservation

**Figure 3:**
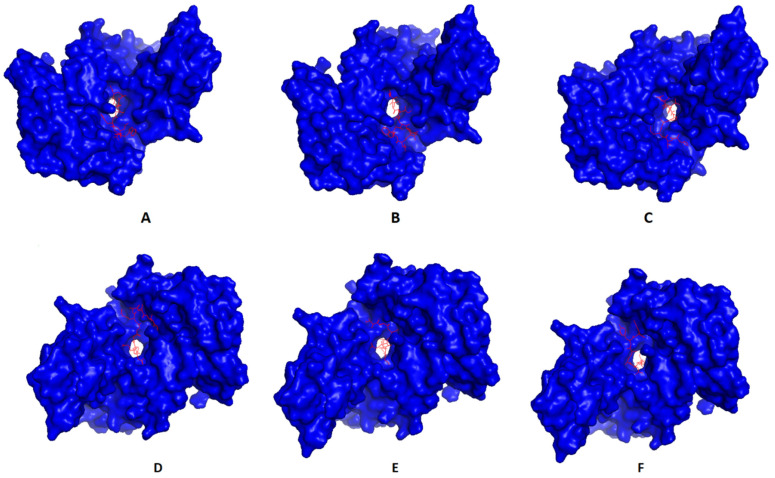
Predicted positions of MHC-I peptides (red color) in the 3D structure of HLA-A*11:01 receptor (blue color), structures (**A**–**F**) are for epitopes number 1,2,3,4,5, and 6, respectively, from Table 5.

**Figure 4:**
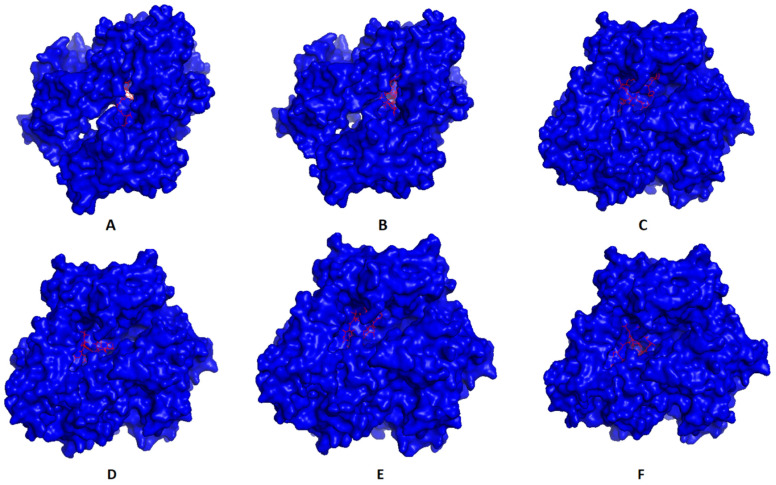
Predicted positions of MHC-II peptides (red color) in the 3D structure of HLA-DRB1*04:01 receptor (blue color), structures (**A**–**F**) are for epitopes number 1,2,3,4,5 and 6, respectively, from Table 5.

Single epitopes were analyzed through a docking study versus a representative allele that acted as a receptor. Figure 3 demonstrates the docking of filtered MHC-I peptides in the receptor of HLA-A*11:01. Figure 4 demonstrates this in MHC-II peptides in the receptor of HLA-DRB1*04:01. The binding energy scores for both types of docking ranged between −7.4 and −9.0 (Table 5), and to validate these scores, we followed the approach mentioned in [60]. Each of the mentioned receptors was deposited in the protein databank with an attached ligand (we employed them to act as a control) that was removed before the docking study and docked again using the same conditions of predicted epitopes docking. The docking score for these controls were −6.3 and −7.7 for HLA-A*11:01 and HLA-DRB1*04:01, respectively. The docking scores of filtered peptides were more negative than the control; therefore, they were estimated to be good binders. Moreover, a comparison of the epitopes’ docking score with that of designed epitopes through a similar approach in studies [61,62,63] demonstrated a more negative docking score in the current study, which also supports the current study’s epitopes to be good binders to their respective receptors.

The epitopes that passed all the previous selection criteria, as seen in Table 5, were analyzed through BLASTp for their conservancy in various mucormycosis-causing fungi and were found to be conserved in a high percentage (Table 6); therefore, they were selected to construct the multitope vaccine.

### 3.5. Assembly of the Multitope Vaccine and Assessment of Its Physicochemical Properties

Filtered single epitopes from CotH and SP were assembled to design the multitope vaccine. We selected 6 CTL epitopes, 6 HTL epitopes, and 4 BCL epitopes and connected them with GGGS, GPGPG, and KK amino acids linkers respectively. Following that, the sequences of PADRE peptide and β-defensin adjuvant were added to generate a multitope vaccine of 337 amino acids with the following sequence:

“EAAAKGIINTLQKYYCRVRGGRCAVLSCLPKEEQIGKCSTRGRKCCRRKKEAAAKAKFVAAWTLKAAAGGGSGQNGRFIWLGGGSRLIQIDVQWGGGSHTMAPLVSFGGGSNDFGGRATWGGGSISSRKALTLGGGSHVAGLAAYFGPGPGAVGRLRLGANLGYLGGPGPGDQFGLLNNIARRPLVGPGPGTDYLSTVNQSLSGFVGPGPGEAVRGSYIVVLKDHLGPGPGDGIKVYVIDTGINVSGPGPGDHAEWISSMVAAKAYKKVFGNDQPGYKRKKPTVKDYIEPRVNKKETPNFKGYAGRKKNYDANTAGDGKKAKFVAAWTLKAAAGGGS”

**Figure 5:**
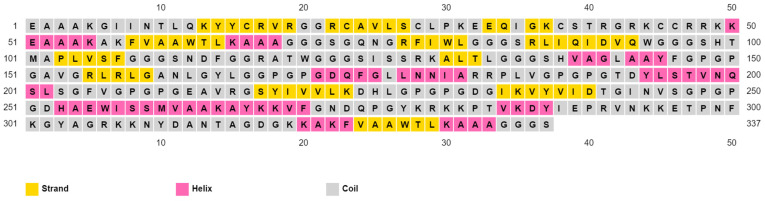
PESIPRED server output for the multitope vaccine secondary structure estimation.

The multitope vaccine sequence started with EAAAK; then, the adjuvant sequence was followed also by EAAAK, and then the PADRE peptide. Following that, the top CTL epitopes for each protein then the top HTL epitopes and BCL epitopes were added and connected with their respective linkers. Finally, a sequence of PADRE peptides was added again. The constructed vaccine assessments showed that it is non-allergen, non-toxic, and antigenic, with an antigenicity score of 1.5, and soluble upon overexpression, with a SOLpro score of 0.95. The vaccine was also analyzed for other physicochemical features, as shown in Table 7. Lastly, the vaccine secondary structure estimation showed 19.29% helix, 20.77% strand, and 59.94% coil (Figure 5).

### 3.6. Vaccine’s Predicted 3D Structure and Its Validation

**Figure 6:**
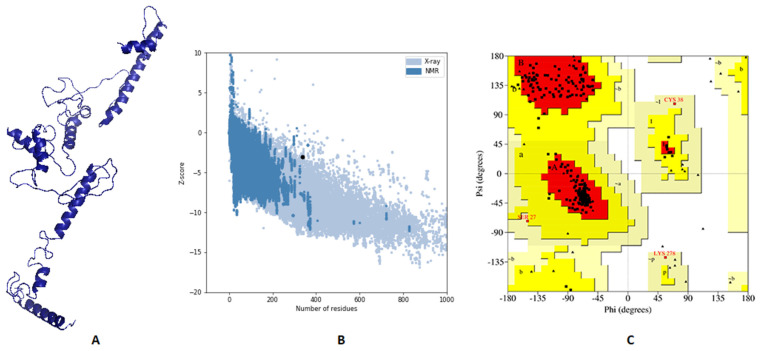
Assessment of the predicted vaccine 3D structure. (**A**) The proposed vaccine structure after refinement; (**B**) A black point demonstrates the predicted Z-score; (**C**) Ramachandran plot analysis of the refined vaccine.

3Dpro webserver generated a 3D structure for the multitope vaccine from the submitted sequence. The validation of this primary structure, via Ramachandran plot analysis and Z-score, showed 88.5%, 11.1%, and 0.4% of residues located in favored, allowed, and outlier regions, respectively, and −2.53 as a Z-score value. Based on these values, we performed structure refinement for that primary structure with the GalaxyRefine server, and the refined model (Figure 6A) exhibited an improvement of its Z-score from −2.53 to −3.07 (Figure 6B). In addition to that, Ramachandran plot analysis also experienced an improvement, where 94.4% and 5.6% of residues were in favored and allowed regions, respectively (Figure 6C).

### 3.7. Vaccine Disulfide Engineering

Disulfide engineering was carried out to generate a protein with better stability. The results of disulfide engineering demonstrated that 31 pairs of amino acids could make disulfide bonds. On the other hand, considering the accepted range of energy (less than 2.2) and Chi3 value (between −87: +97) gave us one recommended pair (SER27-ALA53) for mutating with cysteine.

### 3.8. Molecular Docking of the Vaccine with TLR2

**Figure 7:**
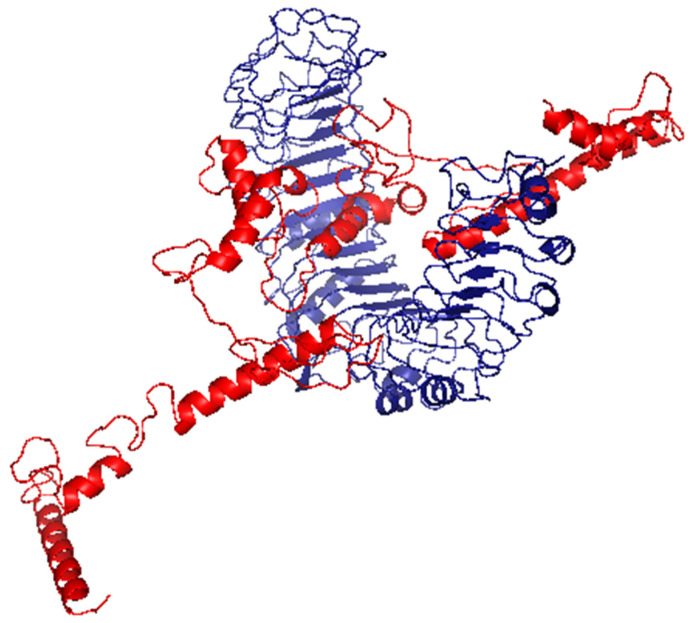
The generated complex of the vaccine ligand (red color) and TLR2 receptor (blue color).

To analyze the affinity between the designed multitope vaccine and TLR2, the ClusPro 2.0 server was employed for a docking study. The server generates 30 predicted complexes with an average docking score of −982.3, and model number 3 (Figure 7) demonstrated the smallest possible binding energy (−1305.7). In order to validate this value, the outer membrane protein (OmpU) from *Vibrio cholera*, which acts as an agonist for TLR-2 [64], was docked with the same server to TLR-2, where 30 predicted complexes were also generated with an average docking score of −1294.3, and the complex with the least docking score was −1471.3. Based on that, the binding energy of the currently designed vaccine on TLR-2 comes between the average and the smallest binding values of an agonist; therefore, a good binding is predicted for the multitope vaccine.

### 3.9. Inherited Flexibility Analysis Using Normal Mode Analysis within Dihedral Coordinates

**Figure 8:**
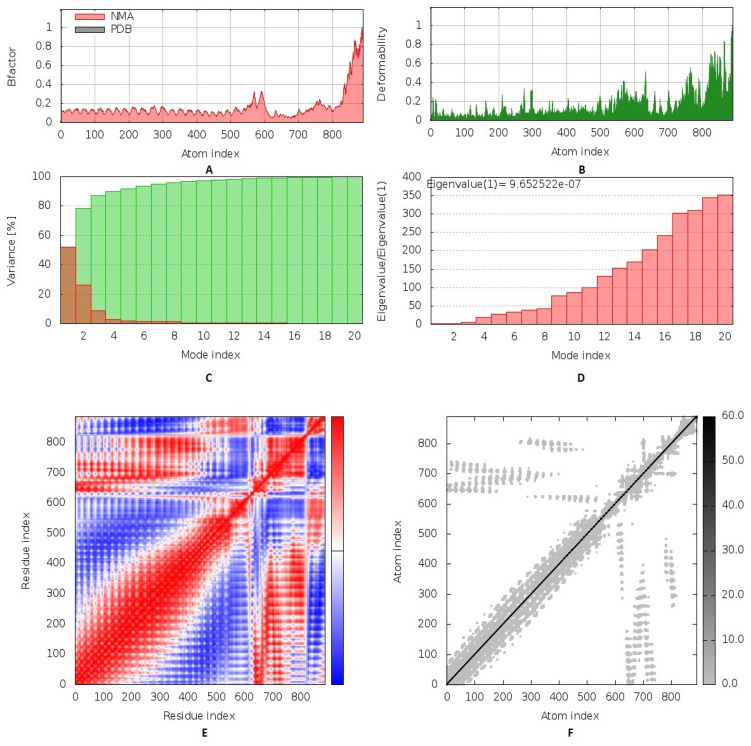
Inherited flexibility analysis of the predicted vaccine–TLR2 complex using normal mode analysis within dihedral coordinates. Complex mobility and flexibility were assessed through (**A**) B-factor values, (**B**) deformability, (**C**) variance, (**D**) eigenvalue, (**E**) covariance of residue index, and (**F**) elastic network analysis.

The collective functional motions/flexibility of the designed multitope vaccine/TLR-2 complex was assessed using the iMODS server. The complex atoms and residues were continuously indexed, where the atom numbers 1–8800 and 8801–13,728 represented those of TLR-2 (first 1–548 residues) and the vaccine (subsequent 1–337 residues), respectively. The B-factor scores of normal mode analysis in the iMODS server indicate the relative amplitude of the atomic displacements around the equilibrium conformation. The values were higher for the vaccine, particularly at the C-terminus, as compared to those of the TLR-2 (Figure 8A). Similarly, the deformability of the complex recapitulates the B-factor findings, where the individual distortion of each vaccine residue was higher than TLR-2, particularly at the C-terminal end (Figure 8B). The estimated eigenvalue of the complex was found to be 9.65 × 10^−7^, while an inverse relationship was found between the eigenvalue and the variance related to each normal mode, predicting significant mobility for the vaccine/TLR-2 complex across collective functional motions (Figure 8C,D). The covariance matrix explained the coupling between pairs of residues, where different pairs demonstrated correlated (red), anti-correlated (blue), or uncorrelated (white) motions. Higher correlated residue–pair motions were assigned for the TLR-2, while more anticorrelated motions were predicted for those of the vaccine protein (Figure 8E). The elastic network model further described the differential flexibility pattern between the TLR-2 and the vaccine (Figure 8F). Continuous dark gray bands were assigned to the TLR-2 residues around the normal distribution stiffer strings, whereas the vaccine residues depict scattered discontinuous gray bands around the normal string of immobility.

### 3.10. Molecular Dynamics Simulations

**Figure 9:**
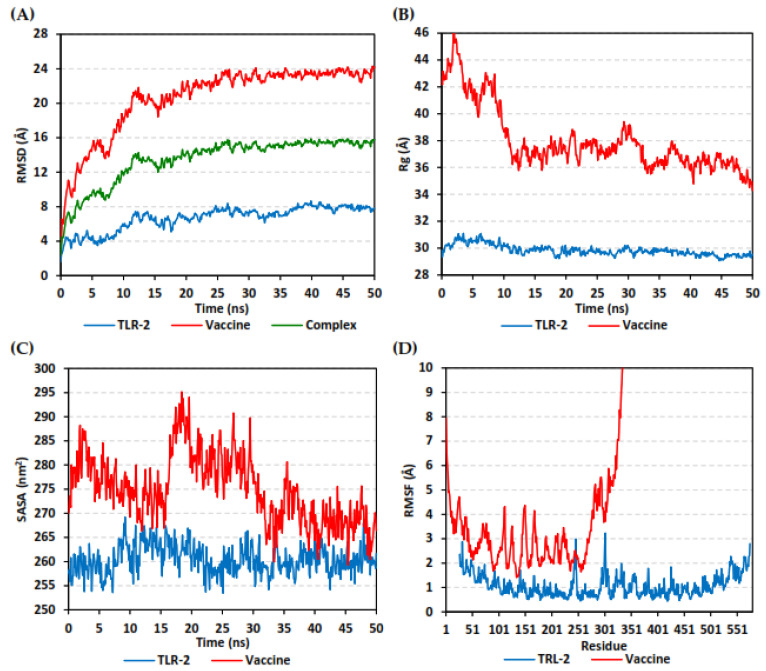
Stability analysis of generated trajectories for investigated multitope vaccine in complex with TLR-2 protein along 50 ns all-atom MD simulation. (**A**) Cα-RMSD, (**B**) Rg, (**C**) SASA, and (**D**) Cα-RMSF trajectories, across MD simulation time (ns).

The estimated RMSD deviations for each protein, in reference to its respective alpha-carbon (Cα), depicted an overall typical behavior for MD simulations (Figure 9A). Over the initial frames, the protein’s RMSD tones increased as a result of the constraint release at the beginning of MD simulation runs. Following the first 20 ns of the MD runs, steady protein’s RMSD trajectories were obtained for more than half of the simulation run time (>25 ns). Notably, the multitope vaccine leveled off at higher RMSD trajectories (23.37 ± 0.41 Å) as compared to the TLR-3 (7.67 ± 0.49 Å) across each respective trajectory plateau and until the end of the MD simulation courses. The RMSD fluctuations were monitored for the combined ligand–receptor complex in reference to the protein backbone’s initial frame, illustrating the Cα-RMSD plateau (15.29 ± 0.32 Å) around 20 ns and until the end of the MD simulation (Figure 9A).

The global stability of the ligand–protein ternary structures was further investigated through monitoring both the Rg and SASA trajectories of the complex entities across the entire MD simulation timeframes. In this study, the steadiest Rg trajectories were assigned for the TLR-2 receptor, showing an average value of 29.84 ± 0.39 Å (Figure 9B). Concerning the multitope vaccine, the protein seemed to be expanding at the initial MD simulation frames, showing the highest Rg tones (max values 46.15 Å). However, the ligand–protein achieved respective compactness and a significant contraction following the 10 ns and until the end of the MD simulation time courses, with average Rg values of 36.90 ± 0.93 Å. On the other hand, the calculated SASA tones for TLR-2 receptor exhibited steady and lower values, 260.42 ± 2.95 nm^2^ (Figure 9C). The vaccine’s SASA trajectories recapitulated the Rg findings. Following the 20 ns MD timeframe, the protein experienced higher fluctuations across the MD simulation run. Nevertheless, almost steady SASA tones were achieved around the 30 ns and until the end of the MD simulation, which was at values slightly higher than those of TRL-2 (268.85 ± 3.77 nm^2^ versus 260.00 ± 2.40 nm^2^).

The fluctuation of each protein’s residues was analyzed by predicting the RMSF stability validation parameter, being able to highlight the residue-wise contribution within the ligand–receptor protein stability. Since investigating the RMSF trajectories for a trajectory region were considered to be stable and the above protein’s RMSD analysis showed significant conformational stability along the 50 ns MD simulations, the Cα-RMSF calculations were reasoned to be estimated across the whole MD simulation trajectories. Notable, the free terminals’ residues of the TLR-2 receptor showed a higher fluctuation pattern (high RMSFs) in comparison to those for the core residues, which is typical for a well-behaved MD simulation. Only two core residue regions; 240–249 and 294–305, showed max RMSF values of 3.09 Å and 3.23 Å, respectively, which correspond to β-loops at the TLR-2′s convex surface (Figure 9D). On the other hand, the vaccine RMSF showed higher values, with more fluctuating tones for its constituting residues (4.29 ± 1.59 Å). Interestingly, higher mobility patterns were depicted for the vaccine’s residues at and vicinal to the C-terminus (high residue ID numbers) as compared to those located near the amine end.

**Figure 10:**
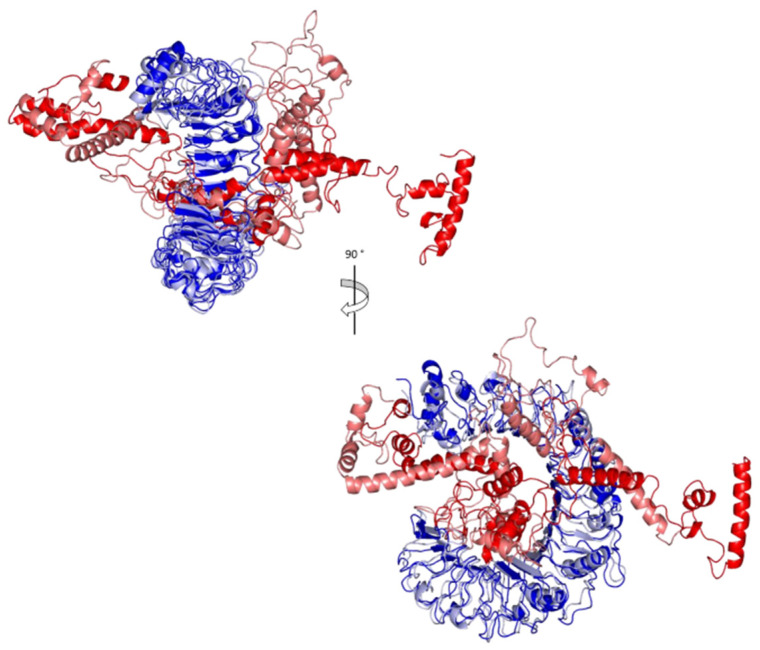
Conformational analysis of MD-simulated multitope vaccine/TLR-2 complex. Overlaid snapshots of the ligand–protein complex at 0 ns and 50 ns of the MD simulation runs. The vaccine and TLR-2 proteins are represented in red and blue cartoon 3D-representation, respectively, where the initial and last extracted frames are obtained at 0 ns (dark colors) and 100 ns (light colors).

Analysis of key conformational alterations for the MD-simulated vaccine and TLR-2 was performed by examining the ligand–protein models at the trajectories in the start and final MD simulation timeframes. Frames at 0 and 50 ns for each ligand–protein model were extracted and minimized to a 0.001 Kcal/mol.A^2^ gradient using MOE (Molecular Operating Environment) system preparation package. A stable binding profile was assigned for the vaccine, showing more compacted anchoring towards the TLR-2 pocket at the end of the MD simulation (Figure 10). Minimal conformational changes were assigned for the TLR-2 protein structure, whereas the vaccine exhibited dramatic alterations regarding its conformation/orientation at the binding site. The C-terminus of the vaccine showed a significant shift from being extended to exhibit closer orientation near the TLR-2 lateral side, where the latter has been reported relevant as the dimerization interface between TLR-2 and the crystallized TLR-1 mediating heterodimerization through major hydrophobic and relevant polar binding interactions.

**Figure 11:**
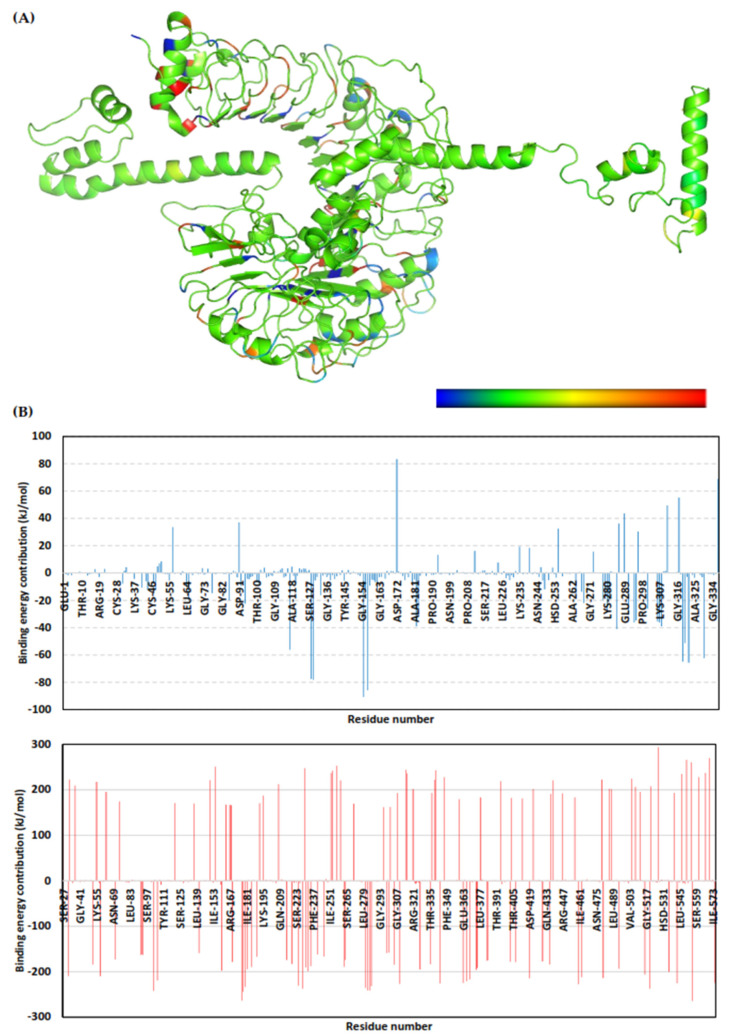
Binding-free energy/residue decomposition illustrating the protein residue contribution at ligand–protein complex ΔG_Total binding_ calculation. (**A**) Cartoon 3D representation for regions of the vaccine/TLR-2 complex favoring binding chemistry. Protein regions are colored in spectrum from dark blue (highly favored with high attractive forces as negative ΔG kJ/mol values) down to dark red (most unfavored with high repulsive forces as positive ΔG kJ/mol values); (**B**) Residue-wise free-binding energy contribution for vaccine (upper panel) and TLR-2 (lower panel) in terms of residues’ sequence numbers.

The multitope vaccine exhibited total free-binding energy towards the TLR-2 binding site. The dissected energy contributions of the van der Waal and electrostatic binding potentials as well as the solvation and SASA energy terms were provided and calculated as kJ/mol ± SD within Table 8. The SASA-only model of the free-binding energy calculation (ΔG_Total_ = ΔG_Molecular Mechanics_ + ΔG_Polar_ + ΔG_Apolar_) was adopted across the 50 ns MD simulation time course, as the complex Cα-RMSDs rapidly attained equilibration/convergence following the few initial MD frames. The decomposition of ΔG_Total binding_ on a per-residue basis identified amino acid residues favoring the vaccine’s binding towards the TLR-2 pocket, where the more negative is the better (Figure 11).

### 3.11. Vaccine Reverse Translation and Codon Optimization

The JCat server was employed for reverse translation and codon optimization for the designed vaccine. The server measured GC content, which was 53.5% (the accepted range is between 30% and 70%). The server also calculated the Codon Adaptation Index (CAI), which was 0.96 (the accepted range is between 0.8 and 1), providing a high probability of protein expression in wet-lab experiments.

### 3.12. Immune Simulation of the Designed Vaccine

**Figure 12:**
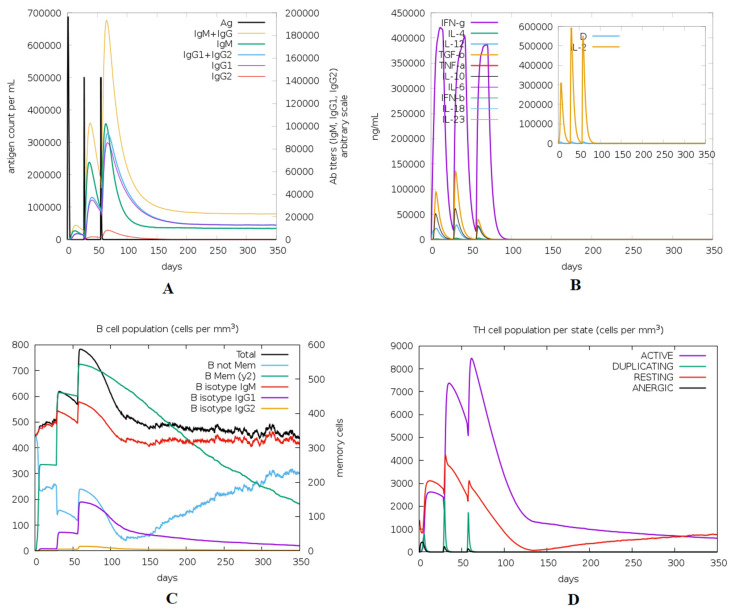
Immune response predicted through ImmSim server after the injection of the designed multitope vaccine. (**A**) Levels of the produced antibodies. (**B**) Cytokines level, (**C**,**D**) demonstrate the B and T cell population respectively.

The immune response regarding antibody titer, cytokines level, and B and T cell population is shown in Figure 12. The multitope vaccine induced the production of high levels of IgM + IgG, and these levels increased with successive injections. Moving to the generated cytokines, several ones were stimulated where INF-γ showed the highest level of induced cytokine. Finally, both B and T cell populations showed a high increase with successive vaccine doses, where the highest level of active B and T cells were shown after the second booster dose injection.

## 4. Discussion

Recently, there has been a revolution in the field of vaccine development as a result of the great progression in bioinformatics, structural biology, and computational tools that have aided largely in the process of handling and analyzing the genomic data of several microorganisms [65]. The approach of predicting and designing vaccines through in silico studies has improved massively in the last few years, where its applications have extended to involve bacteria, viruses, fungi, and even cancer [66]. The current days of the COVID-19 pandemic stressed our need to develop effective management approaches to control opportunistic infections and protect immunocompromised patients [67], Therefore developing an effective vaccine against mucormycosis is a major health priority. The usage of in silico approaches in designing and validating vaccines computationally can save both time and cost. This can be explained by considering the microorganisms that are difficult to be cultivated or infections that are caused by a group of microorganisms, such as mucormycosis; computational tools can save time and analyze the proteome of these types of microorganisms and detect potential vaccine candidates. In addition to this, mapping the epitopes of these candidates and performing a docking analysis with their respective receptors can give an overview of the behavior of these epitopes when they encounter human immune receptors. Therefore, computational tools can make a primary validation before moving to the costly lab experiments, which is certainly an economic approach. Due to these advantages, the approaches of reverse vaccinology and immunoinformatics, based on computational vaccine designing and analysis, have been applied in many studies during the last few years and have shown promising results, which provides a practical validation of computational prediction methods when the studies applied wet-lab experiments on the designed vaccine [15,21,68,69,70].

The current study started by analyzing the virulent proteins of major mucormycosis-causing fungi. The selection of these proteins to be our primary vaccine candidates for the multitope design relied on major factors. Firstly, the protein must contribute to the fungal virulence; hence, the primary list was created after studying mucormycosis virulent protein in the literature [19]. The second factor was the antigenicity score of filtered proteins as the protein candidate must be antigenic. The third factor was the subcellular localization of the selected proteins, where adhesins that have a role in binding to the infected host tissue [71] and secreted proteins that have a role in tissue penetration and fungal nutrient acquisition [72] are considered potential targets for designing an effective vaccine against invasive fungi.

After applying the major filtration factors, we came up with serine protease (SP) and spore coat protein (CotH) to be the vaccine candidates of the current study. Serine proteases are essential hydrolytic enzymes that use catalytic serine residue for breaking peptide bonds in proteins. Fungi use this type of enzyme for nutrient breakdown and acquisition from protein-rich sources [73]. In addition to this, serine protease can be utilized for the protection of the fungal cell from the host’s immune system by degrading chitinases that target the fungal cell wall [74]. CotH protein is universally present in Mucorales and has a significant role in binding to and invading host epithelial cells [75]. The potential selection of CotH as a vaccine candidate against Mucorales was estimated in [76], and it was found that specific antibodies for glucose-regulated protein 78/CotH interactions decrease the injury of endothelial cells as a result of Mucorales infection and protect mice from mucormycosis.

Protein extraction through wet-lab methods, in growing fungi and validating these proteins as vaccine candidates, is considered a costly time-consuming process; thus, the application of immunoinformatics tools that can recommend potential candidates, before wet-lab experimental validation, will save costs and time. Furthermore, epitope mapping via immunoinformatics tools will exclude the non-antigenic regions of protein candidates, where only epitopes that stimulate B and T cells would be selected for the vaccine design, which would give a more potent immune response [77]. The approach of designing and validating a vaccine through a computational approach has been applied against many fungi such as *Aspergillus fumigatus* [78], *Candida auris* [79], and *Candida albicans* [18]. This approach had been validated with wet-lab experiments, and immunized mice were protected from fatal candidiasis [80]. To the best of our knowledge, this is the first study that designed and validated a vaccine against mucormycosis based on immunoinformatics and computational tools.

In the current study, we mapped B- and T-cell epitopes for SP and CotH proteins, and the generated epitopes were filtered according to the percentile rank, antigenicity score, the number of reacting alleles, and the binding energy with a respective receptor. Top-ranked epitopes, beta-defensin adjuvant, and PADRE peptide were assembled to constitute a multitope vaccine with specific activity against mucormycosis-causing fungi and reduced HLA polymorphism in the population [81]. Generally, multitope vaccines have the advantage of being more efficient than single epitopes [63]. As we mentioned, mucormycosis is caused by various types of fungi, and it would be too difficult to find a single epitope with high conservation in those fungi; therefore, the multitope construct offers a putative solution by combining several epitopes, each of them with a high conservation percentage, in some of the mucormycosis-causing fungi. After investigation of the conservancy of every single epitope that constructs the current study’s multitope vaccine, in seven major mucormycosis-causing fungi, we found that at least two single epitopes were 100% conserved in each fungus, which recommends the current study’s multitope vaccine as a general vaccine against mucormycosis. A molecular docking study between the designed vaccine and TLR2, which is involved in the recognition of Mucorales [82], was performed, and the stability of the docked complex was assessed through molecular dynamics simulation.

Throughout the 50 ns all-atom MD runs, the multitope vaccine illustrated significant global stability within the target’s canonical binding site, confirmed through the monitored RMSD trajectories. The estimated *Cα*-RMSD deviations for each protein illustrated conventional thermodynamic behaviors across the MD simulation runs. Leveling off over more than 25 ns indicated the successful convergence of both proteins across the designated MD simulation timeframe. Monitoring the RMSD fluctuations for the combined ligand–receptor complex in reference to the protein backbone initial frame ensured the ligand’s confinement within the TLR-2 canonical binding site across the MD run. The latter came in great concordance with several reported multitope proteinaceous vaccines against the TLRs of different microorganisms, where their respective MD simulation studies illustrated preferential vaccine stability at the receptor’s binding sites over 10-nanosecond or 20-nanosecond timeframes [83,84,85]. Notably, the vaccine–receptor complex described within the presented study showed significant stability over a longer timeframe of a 50-nanosecond MD simulation run.

The higher RMSD for the vaccine suggested dramatic conformational changes for the vaccine structure until reaching an equilibration stage and convergence following the 20-nanosecond window. This was confirmed through the latter Rg and SASA trajectory analysis, where these parameters provided great insights regarding the global stability of the ligand–protein ternary structures. Typically, the estimated radii of gyration of the investigated complexes permitted the exploration of the complex rigidity and compactness, where this stability parameter accounts for the complex’s mass-weighted root-mean-square distance relative to its common mass center. In these regards, low Rg values achieving a plateau around an average value would be correlated to the sustained stability/compactness of an investigated complex [86]. On a similar basis, decreased SASA tones imply relative structural shrinkage for the ligand–protein complexes under the impact of the solvent surface charges, yielding more compact and stable conformations. The latter has been correlated to the SASA calculation, which estimates the molecular surface area being assessable to solvent molecules, providing a quantitative measurement of the complex–solvent interaction [87]. The higher Rg and SASA tones at the initial frames were correlated to the expanded and more extended vaccine conformation at the beginning of the MD simulation run. However, significant compactness as well as favored inter- or intra-molecular interactions between the vaccine and TRL-2 was suggested, since the vaccine finally attained lower steady tones in the final run. Additionally, the relatively small SASA differences for both the vaccine and the receptor conferred preferential ligand confinement within the TLR-2 binding site, since ligand–receptor binding is considered a solvent-substitution process.

The above vaccine’s initial higher fluctuating dynamic behavior was generally expected and significantly rationalized to the inherited folding/packing of its tertiary protein structure. Exhibiting extended *α*-helices with long connecting flexible *β*-loops at the beginning of the MD simulation run suggested significant protein relaxation and final convergence into a more compacted and stabilized conformation. In comparison to much lower RMSD, Rg, and SASA values, the TLR-2 receptor maintained its highly compacted shoe-like architecture of highly ordered parallel *β*-sheets from the beginning until the end of the MD simulation run. The latter comparative flexibility was also depicted through the obtained inherited flexibility analysis using normal mode analysis within dihedral coordinates, where higher mobility, deformability, and B-factors were assigned for the vaccine as compared to TLR-2. Stiffness and immobility profiles across the TLR-2 residues that were obtained from the covariance of residue index and elastic network analysis showed uniform bands towards the normal stiffness strings, which was not the same for those of the vaccine. The latter suggested greater inherited flexibility for the vaccine protein. Further insights regarding this vaccine/TLR-2 comparative conformational evolution across the MD simulation were illustrated through the estimated RMSF trajectories across the 50-nanosecond runs and conformational analysis for the initial and final MD simulation frames. Having low RMSF tones across most of the TLR-2 residue regions conferred the significant influence of the vaccine’s binding upon the stability of TLR-2, or in other terms, the pivotal role of these TLR-2 residue ranges for the stability of vaccine within its respective binding site [88]. These findings were also consistent with the above-reported studies investigating the potential binding affinity of peptide-based vaccines towards microorganism TLRs [83,84,85]. The higher RMSF values for the vaccine further highlight the lower comparative intramolecular interactions among its respective residues compared to TLR-2, based on their protein folding/packing. The high immobility profiles for the vaccine’s C-terminal residues confer significant conformational changes for this protein side for attaining more stable and final compact architecture, which was clearly illustrated through the performed conformational analysis. Having the C-terminus of the vaccine binding with a close orientation at the TLR-2 lateral side can suggest the potential impact of the vaccine for hampering the reported TLR-2/TLR-1 heterodimerization, which is significant and essential for recognizing bacterial lipoproteins and lipopeptides [89]. The latter further ensures the capability of the vaccine in not only blocking the TLR-2 pocket against bacterial lipoproteins/lipopeptides anchoring but also in further interfering with the association between TLR-2 and TLR-1, suggesting a synergistic effect for minimizing cellular responsiveness against bacterial antigens.

The MM/PBSA calculation was implemented for a binding-free energy estimation [57]. To our delight, the multitope vaccine depicted significant free-binding and affinity towards the TLR-2 binding’s pocket. Dissecting the obtained binding-free energy into its contributing energy terms showed a dominant energy contribution of the electrostatic interactions over the van der Waal potentials within the free-binding energy calculation. However, the total non-polar interactions (Δ*G*_van der Waal_ plus Δ*G*_SASA_) confer a large surface area of the TLR-2 pocket, as well as being reasonably satisfactory to counterbalance the predicted electrostatic penalties and solvation energies during ligand binding. The latter was rationalized since the reported data within the current literature have considered the TLR-2 pocket to be more hydrophobic in nature [89,90,91]. Finally, the high solvation energies, which represent significant repulsive forces against the ligand binding, were suggested to be related to the extended vaccine surface being exposed to the solvent front. These large repulsive forces were mediated majorly by the TLR-2 residues rather than by the vaccine amino acids as being depicted within the residue-wise energy contribution, which could be related to the high ordered water molecules at the hydrophobic surface of the TLR-2 ligand-binding site.

## 5. Conclusions

The current study shows the advantages of bioinformatics tools in designing a potential vaccine against mucormycosis-causing fungi. The proteome investigation process came up with two protein candidates that were shared between several mucormycosis-causing fungi. Epitope mapping generated a pool of B- and T-cell epitopes that were assembled to constitute a multitope vaccine. This vaccine showed promising immunological and physicochemical characteristics. The multitope vaccine-receptor docking study with a detailed investigation of the docked complex through molecular dynamics simulation in addition to the computationally predicted immune response for the injected vaccine recommended that the current study design vaccine as a putative solution against mucormycosis. Wet lab validation is required in future studies to validate our findings.

## Figures and Tables

**Table 1:** Antigenicity and cellular localization of virulent proteins in *Rhizopus microsporus*.

Protein Name	Antigenicity Score	Surface Exposed or Secreted Protein
High-affinity iron permease	−0.03	-
Spore coat protein	0.80	Yes
Serine protease	0.86	Yes
Calcium/calmodulin-dependent protein kinase	0.55	No

**Table 2:** Predicted B cell epitopes from CotH and SP proteins.

CotH			SP		
Epitope	Start–End	Antigenicity Score	Epitope	Start–End	Antigenicity Score
CATDPSYI	38–44	1.14	YAPVEAEAV	36–44	−0.11
VFGNDQPGYKR	109–119	2.04	ETPNFKGYAGR	96–106	2.04
PTVKDYIEPRVN	148–159	1.1	NYDANTAGDG	161–170	1.69
QEYPSKSVSKDHT	166–178	0.54	IAGTKYGVAKKARP	217–230	1.42
LVPANEQKDADNSFK	265–279	−0.09	SNGSGSMSD	238–246	0.12
			KDKEQAQTEGKPFKG	260–274	0.84
			NTATNTISGTSMASP	364–378	1.5
			QSEPGVTPKEI	390–400	0.35
			PNELTKIPKDT	410–420	−0.98

**Table 3:** Filtered MHC-I peptides of CotH and SP proteins.

CotH			SP		
Epitope	Antigenicity	Reacting Alleles Number	Epitope	Antigenicity	Reacting Alleles Number
GQNGRFIWL	2.96	7	TAGDGIKVY	4.3	9
RVFGNDQPGY	2.63	11	NDFGGRATW	2.89	7
ARASYVRLF	2.26	6	ISSRKALTL	2.62	6
RLIQIDVQW	2.02	12	ATWGKTIPA	2.6	8
TVNQSLSGF	1.91	7	HNDFGGRATW	2.59	6
YQDPGQNGRF	1.91	14	HVAGLAAYF	2.4	12
VQWDKQLQR	1.68	9	APGLDIQSIW	2.19	6
RIMQDYYDY	1.63	8	SSRKALTLR	1.96	6
HTMAPLVSF	1.35	19	VVLKDHLSM	1.23	11
SQLLQVDEF	1.41	10	KARPVAVKV	1.11	11

**Table 4:** Filtered MHC-II peptides of CotH and SP proteins.

CotH				SP			
Epitope	Antigenicity	IFN Epitope	Reacting Alleles	Epitope	Antigenicity	IFN Epitope	Reacting Alleles
AVGRLRLGANLGYLG	1.66	Yes	4	EAVRGSYIVVLKDHL	1.77	Yes	9
KIKFSLSGQTSRLFN	1.73	No	6	DGIKVYVIDTGINVS	1.64	Yes	9
VGRLRLGANLGYLGP	1.8	Yes	4	GIKVYVIDTGINVSH	1.39	Yes	8
DQFGLLNNIARRPLV	0.99	Yes	8	LARISSRKALTLRNF	1.02	Yes	7
DYLSTVNQSLSGFVL	1.46	Yes	4	DHAEWISSMVAAKAY	0.71	Yes	13
FGLLNNIARRPLVSQ	1.07	Yes	4	AITVGASTIADERAY	0.69	Yes	5
TDYLSTVNQSLSGFV	1.68	Yes	6	QDHAEWISSMVAAKA	0.68	Yes	10
GRLRLGANLGYLGPT	1.56	Yes	4	HAEWISSMVAAKAYN	0.58	Yes	12
ITDYLSTVNQSLSGF	1.68	Yes	5	GDGIKVYVIDTGINV	2.63	No	9
QFGLLNNIARRPLVS	1.07	Yes	7	GSYIVVLKDHLSMEQ	2.21	No	8

**Table 5:** Selected epitopes’ binding energies with representative MHC-I and MHC-II alleles.

No.	Epitope	MHC-I Allele	Binding Energy (kcal/mol)	Epitope	MHC-II Allele	Binding Energy (kcal/mol)
1	GQNGRFIWL		−7.8	AVGRLRLGANLGYLG		−8.1
2	RLIQIDVQW		−7.4	DQFGLLNNIARRPLV		−8.0
3	HTMAPLVSF	HLA-A*11:01	−8.3	TDYLSTVNQSLSGFV	HLA-DRB1*04:01	−7.6
4	NDFGGRATW		−8.8	EAVRGSYIVVLKDHL		−7.6
5	ISSRKALTL		−8.0	DGIKVYVIDTGINVS		−7.7
6	HVAGLAAYF		−9.0	DHAEWISSMVAAKAY		−8.4

**Table 6:** Conservancy analysis of top-ranking epitopes in several mucormycosis-causing fungi.

				Percentage Identity (%) in				
Epitope Sequence	Epitope Type	*Rhizopus microsporus*	*Rhizopus azygosporus*	*Rhizopus oryzae*	*Rhizopus delemar*	*Mucor lusitanicus*	*Apophysomyces* sp. BC1015	*Lichtheimia corymbifera*
GQNGRFIWL	CTL	100	100	87.5	75	75	83.33	100
RLIQIDVQW	CTL	100	100	75	87.5	83.33	100	100
HTMAPLVSF	CTL	100	100	100	100	70	100	72.73
NDFGGRATW	CTL	100	75	87.5	87.5	87.5	87.5	77.78
ISSRKALTL	CTL	100	100	80	88.89	75	75	85.71
HVAGLAAYF	CTL	100	88.89	100	88.89	100	100	100
AVGRLRLGANLGYLG	HTL	100	100	86.76	86.76	73.33	87.50	88.89
DQFGLLNNIARRPLV	HTL	100	100	73.33	73.33	80	100	88.89
TDYLSTVNQSLSGFV	HTL	100	93.33	100	100	100	100	80
EAVRGSYIVVLKDHL	HTL	100	66.67	86.67	80	86.67	100	71.43
DGIKVYVIDTGINVS	HTL	100	78.57	92.86	92.86	85.71	92.86	91.67
DHAEWISSMVAAKAY	HTL	100	71.43	73.33	93.73	83.33	75	77.78
VFGNDQPGYKR	BCL	100	100	77.78	75	85.71	75	87.5
PTVKDYIEPRVN	BCL	100	91.67	69.23	70	75	87.5	80
ETPNFKGYAGR	BCL	100	72.73	81.82	81.82	81.82	87.5	87.5
NYDANTAGDG	BCL	100	75	80	70	80	87.5	75

**Table 7:** Scores of the vaccine’s physicochemical characteristics assessment.

Physicochemical Characteristic	Molecular Weight	TheoreticalpI	Extinction Coefficient	GRAVY	Instability Index	Aliphatic Index
Score	34.78 kDa	10.03	51,255 M^−1^ cm^−1^	−0.326	23.82	70.42

**Table 8:** Total binding-free energies and individual energy term (ΔG_Total binding_ ± SD) concerning the designed multitope vaccine at TLR-2 protein binding site.

Energy (kJ/mol ± SD)	Ligand–Receptor Complex
ΔG_van der Waal_	−880.969 +/− 125.941
ΔG_Electrostatic_	−2732.441 +/− 151.150
ΔG_Solvation; Polar_	2019.511 +/− 137.941
ΔG_Solvation; SASA_	−119.052 +/− 9.876
ΔG_Total binding_	−1712.950 +/− 160.827
